# Beam focal spot position: The forgotten linac QA parameter. An EPID‐based phantomless method for routine Stereotactic linac QA

**DOI:** 10.1002/acm2.12147

**Published:** 2017-08-08

**Authors:** Jacek M. Chojnowski, Michael P. Barnes, Jonathan R. Sykes, David I. Thwaites

**Affiliations:** ^1^ Department of Radiation Oncology Coffs Harbour Base Hospital Coffs Harbour NSW Australia; ^2^ Institute of Medical Physics School of Physics University of Sydney Sydney NSW Australia; ^3^ Department of Radiation Oncology Calvary Mater Hospital Newcastle Newcastle NSW Australia; ^4^ School of Medical Radiation Sciences University of Newcastle Newcastle NSW Australia; ^5^ Department of Radiation Oncology Blacktown Cancer & Haematology Centre Blacktown NSW Australia

**Keywords:** EPID, focal spot, quality assurance

## Abstract

Modern day Stereotactic treatments require high geometric accuracy of the delivered treatment. To achieve the required accuracy the IGRT imaging isocenter needs to closely coincide with the treatment beam isocenter. An influence on this isocenter coincidence and on the spatial positioning of the beam itself is the alignment of the treatment beam focal spot with collimator rotation axis. The positioning of the focal spot is dependent on the linac beam steering and on the stability of the monitor chamber and beam steering servo system. As such, there is the potential for focal spot misalignment and this should be checked on a regular basis. Traditional methods for measuring focal spot position are either indirect, inaccurate, or time consuming and hence impractical for routine use. In this study a novel, phantomless method has been developed using the EPID (Electronic Portal Imaging Device) that utilizes the different heights of the MLC and jaws. The method has been performed on four linear accelerators and benchmarked against an alternate ion chamber‐based method. The method has been found to be reproducible to within ±0.012 mm (1 SD) and in agreement with the ion chamber‐based method to within 0.001 ± 0.015 mm (1 SD). The method could easily be incorporated into a departmental routine linac QA (Quality Assurance) program.

## 1 INTRODUCTION

Quality Assurance of medical linear accelerators is necessary for safe treatment of radiotherapy patients. Recommendations on tests required with frequencies and tolerances are described in specialized publications such as AAPM TG‐142^1^ and IPEMB81.^2^ Modern linear accelerators have improved accuracy and precision in treatment delivery and allowed clinical implementation of advanced treatment modalities such as frameless Cranial Stereotactic Radiosurgery (CSRS) and Stereotactic Body Radiotherapy (SBRT). These treatment techniques demand more stringent linac quality assurance programs with additional complex checks which lead to increased time and resource demands.^3^

The correct delivery of treatments requiring high spatial accuracy such as CSRS and SBRT means that the geometric accuracy of Image‐Guided RadioTherapy (IGRT) systems must be ensured. Recommendations for IGRT system QA are provided in AAPM TG‐179 report.^4^ This includes the recommendation for testing the imaging and treatment isocenters coincidence. This is often achieved using a Winston Lutz (WL) style measurement.^5^ If the ball bearing to be imaged is placed at the imaging isocenter, then the imaged measured distance from collimator to ball bearing with gantry and collimator rotation can be used to provide a measure of imaging to treatment isocenter coincidence. This measurement provides an overall process type test with several influencing variables, which makes it difficult to diagnose the cause of an unacceptable result. Regular individual testing of each of the influencing variables should ensure stable WL results and provide a methodology for investigation of any WL fails that are observed. The variables that influence the treatment isocenter component of WL include mechanical isocenter size and shape, alignment of collimators and beam focal spot position relative to collimator rotation axis,^6^ and their stability with gantry rotation.

Like the treatment isocenter; the imaging isocenter is also influenced by the mechanical isocenter as the axis of gantry rotation is shared. The geometric calibrations of the IGRT system account for the influences on imaging isocenter and TG‐179 recommends updating these monthly. Of special mention is the Varian IsoCal geometric calibration, which Varian uses to help align the imaging and treatment isocenters.^7^, ^8^ Like the WL measurement, this geometric calibration is influenced by multiple variables and is only performed using the 6 MV beam. Because of potential variability in focal spot position between beam energies due to each beam being positioned and steered separately,^9^ the IsoCal calibration may not ensure coincidence between treatment and imaging isocenters for all beam energies. This indicates the need for a regular check of beam focal spot position if beams other than 6 MV are to be used for CSRS or SBRT treatments.

**Figure 1:**
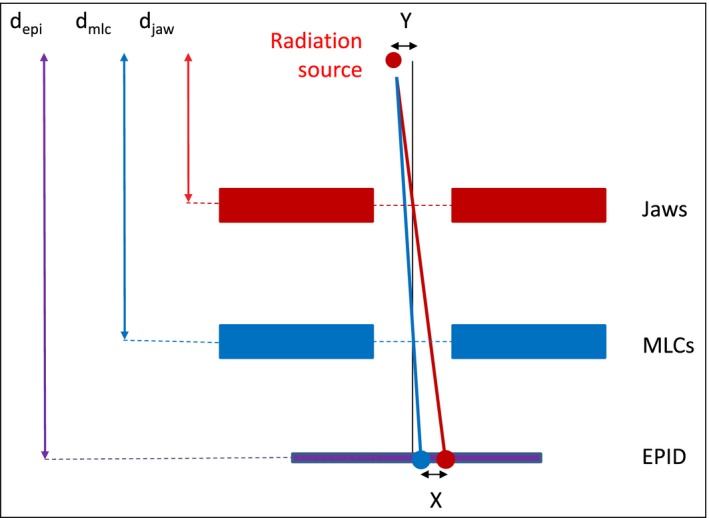
Diagram of a Varian linac head (schematic and not to scale) and illustration of radiation focal spot position determination using the EPID. Vertical black line represents the collimator rotation axis. Red line represents center of jaw defined field with 180° collimator rotation. Blue line represents center of MLC defined field with 180° collimator rotation.

Beam focal spot position is often not tested regularly in radiotherapy departments because it is not explicitly recommended in TG‐142. However, in the interest of ensuring WL stability for CSRS and SBRT treatments and for ensuring coincidence of imaging and treatment isocenters for beams other than 6 MV, its testing is indicated. Furthermore, as an effect of a significant shift in beam focal spot position is a lateral shift of the beam^6^ (Fig. 1), correct focal spot alignment is required for geometric accuracy for all treatment types and is required for constancy of the beam profile, which is recommended by TG‐142. On Varian linacs the position of the focal spot is controlled by the Radial and Transverse position steering servos (Pos R and Pos T). These servos analyze the difference in the signals from the outer “C” sections of the Monitor Chamber at 50 ms intervals (10 ms for Truebeam^®^) and the feedback signal correct the electron beam spot position. This system is calibrated as part of the beam steering process separately for each photon beam energy, such that a balanced servo should equate to a focal spot aligned to collimator rotation axis. Because of the potential for drift in the Monitor chamber response, it is indicated to check the focal spot position on a regular basis if a correctly steered beam is to be assured.

Two accurate and independent methods of measuring focal spot position are described by Nyiri.^10^ The first method, the corotational penumbra modulation measurement, uses an ionization chamber mounted on a jig attached to a collimator near the 50% beam edge position. A modified version of this method will be used in this study as the independent validation technique. The second method, the image center shift method, uses multiple EPID images of two opaque rods attached to a jig at two different geometric distances to the x‐ray source. Both of those methods require a specially made jig which is not suitable for quick and routine measurements.

This study describes a first phantomless method of evaluating focal spot alignment to collimator rotation axis with no additional tools or assumptions necessary. The method presented is robust, accurate, and easy to perform.

## 2 MATERIALS AND METHODS

### 2.A Linear accelerators

All tests were conducted on four linear accelerators at the Crown Princess Mary Cancer Centre (Westmead, Australia), namely: two Varian Clinac^®^ 6EX (Varian Medical Systems, Palo Alto, CA, USA) with the aS500 EPID, one Varian Clinac^®^ 21iX with the aS1000 EPID, and one Varian Truebeam^®^ with the IDU EPID. The sensitive area for all three panels is 40 cm × 30 cm. The EPID aS500 has an array of 512 × 384 pixels and the EPID aS1000 and IDU has an array of 1024 × 768 pixels. Pixel size for the aS500 is 0.784 mm and for both the aS1000 and IDU panels is 0.392 mm, i.e., half the pixel size of the aS500. However, the images acquired on the Clinac^®^ 21iX (in dosimetric mode) are of lower resolution with 512 × 384 pixels and a pixel size of 0.784 mm, the same as for the aS500.

### 2.B Method

The alignment of the focal spot with collimator rotation axes can be determined from beam center measurements from collimators at two different distances (Fig. 1).

In this study the jaws and MLC were used as the collimators. Firstly, the jaws were set to 10 × 10 cm^2^ and 20 MU was delivered at collimator angles 90° and 270°. The beam was imaged using the EPID in integrated mode at the EPID source‐imager distance equal to 100 cm for Varian Truebeam^®^ and Clinac^®^ 21iX and 105 cm for two Varian Clinacs^®^ 6EX. From the image the edge of the field was determined as the position of the 50% intensity points. From these points the position of the center of field was calculated in both inplane (X jaws) and crossplane (Y jaws) directions. The whole process was then repeated with jaws retracted and 10 × 10 cm^2^ MLC defined fields. By averaging the beam centers from the collimator symmetric measurements (i.e., 90° and 270°) the influence of MLC and jaw miscalibration is averaged out and the focal spot misalignment with collimator rotation axis isolated. The magnitude of the misalignment can then be calculated using eqs. (1) and (2) and Fig. 1.

(1) $$D_{\mathrm{RFS}}=a^{*}D_{\mathrm{EPI}}$$

Where:

D_RFS_ = Radiation focal spot offset D_EPI_ = Measured distance between field centers using the EPID a = machine‐ and procedure‐specific proportionality factor.

(2) $$a=\frac{1}{\frac{\left(d_{\mathrm{epi}}-d_{\mathrm{jaw}}\right)}{d_{\mathrm{jaw}}}-\frac{\left(d_{\mathrm{epi}}-d_{\mathrm{mlc}}\right)}{d_{\mathrm{mlc}}}}$$

Where: d_epi_ = distance from the X‐ray target (focal spot) to the EPID d_jaw_ = distance from the X‐ray target (focal spot) to the jaws d_mlc_ = distance from the X‐ray target (focal spot) to the MLC.

### 2.C Image processing algorithm

All acquired EPID images were analyzed by a custom MATLAB^®^ (The MathWorks, Inc., Natick, USA) script to determine the two radiation isocenter centroids defined by the jaws and MLC, respectively. First, each image was filtered to remove noise using two‐dimensional median filtering with a 3 × 3 size matrix. Each image was scaled to pixel values between 0 and 1, consequently the minimum value was assigned the value 0 and the maximum value was assigned the value 1. Each image was then resized, using bicubic interpolation, by a factor of 20 to increase the calculation resolution except for the Truebeam^®^ linac, where each image was resized by a factor of 10, thus the output image pixel spacing was identical for all linacs. Next, each image was converted into a binary image with a threshold equal to 0.5 that relates to the Half‐Value‐Full‐Width of the radiation field to determine the field edges. The center of each field was then calculated as the average point between field edges in both X and Y jaws directions. The radiation isocenter centroid defined by the jaws was then calculated as the average position of all field centers. The same process was performed with the aperture formed by the MLC only.

The distance between the two radiation isocenters defined by the jaws and the MLC at the EPID level was then calculated as the difference between the two isocenters expressed in pixels multiplied by pixel size for a given EPID panel and the resize factor used (10 for the Truebeam^®^ and 20 for the other linacs).

To calculate radiation focal spot offset, the distance determined between the two isocenters was multiplied by the proportionality factor “a” (eq. (1)) specific for the machine and the EPID source‐imager distance. In this study the following parameters have been used: d_mlc_ = 49 cm and d_jaw_ distances are 40.6 cm and 31.9 cm for X and Y jaws, respectively. Therefore, the proportionality factor “a” defined in eq. (2) was equal to 2.368 and 2.2556 for X jaws and 0.9141 and 0.8706 for Y jaws with the EPID source‐imager distance equal to 100 cm and 105 cm, respectively.

### 2.D Reproducibility

The test was executed once per week on each linac over 3 weeks to observe reproducibility.

### 2.E Independent validation

The validation of the phantomless method is based on the work published by Nyiri^10^ with minor modification. The ionization chamber spatial sensitivity was determined by shifting the X and Y jaws, not the jig with the ionization chamber as in the original work.

**Figure 2:**
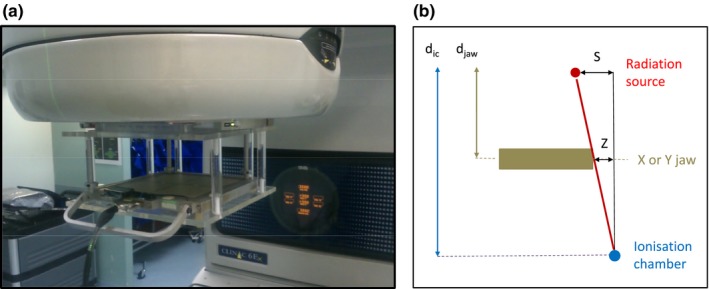
A setup of (a) the dosimetry tool attached to a collimator on a Varian Clinac^®^ 6EX (b). The illustration of obscuring of the focal spot by the jaw and the geometric relationship of the lengths of similar triangles (schematic and not to scale).

The validation procedure was performed using an ionization chamber [a central detector embedded in a “TRACKER” beam evaluation tool (Fluke Biomedical, Everett, WA, USA),^11^ Fig. 2(a)], positioned at the axis of the collimator rotation.

The technique consisted of three distinctive steps:

1. Measurement of the sensitivity of a chamber to small changes in jaw position with a half‐blocked field, i.e., changes in charge collected per 100MU per 1 mm change in either the half‐blocked X or Y jaw position at the isocenter level.
2. Correlate geometrical shift of the jaw at the physical jaws level, i.e., 1 mm shift of X and Y jaw at isocenter level equals 0.406 mm and 0.319 mm shift, respectively, at the physical X and Y jaws position (jaws X and Y are located 40.6 cm and 31.9 cm from the source, respectively).

The charge collected from the chamber depends on the amount the focal spot is obscured by the jaw [Fig. 2(b)]. Therefore, from the chamber point of view being half‐blocked by the jaw, moving a jaw infinitesimally is equivalent to a shift of the source (a first‐order linear approximation). Based on the geometric ratios of lengths of similar triangles, the position of the source is proportional to a shift of either X or Y jaws by:

(3) DRFS=dic(dic−djaw)∗Z

Where: D_RFS_ = Radiation focal spot offset Z = Jaw shift (either X or Y) d_ic_ = distance from the X‐ray target (focal spot) to the ionization chamber d_jaw_ = distance from the X‐ray target (focal spot) to the X or Y jaws.

(The ionization chamber is located 75 cm from the source, hence, D_RFS_ = 1.74*Z for Y jaw and D_RFS_ = 2.18*Z for X jaw.)

Hypothetical shift of a focal spot can directly be correlated with a shift of the X or Y jaw. And, from the measured chamber sensitivity data, a shift of the X or Y jaw can be further correlated with the change in charge measured by the ionization chamber. In the reversed scenario, the focal spot offset can be determined from the measured charge by the ionization chamber.

1. Measure the charge by the half‐beam blocked ionization chamber at two opposite collimator angles, namely, 90° and 270°. If the focal spot is centered at the collimator axis of rotation, then the two charge readings will be the same. However, if the focal spot is offset from the collimator axis of rotation, then this offset is directly proportional to half of the difference between the readings by the ionization chamber. Using both X and Y jaws one can determine the focal spot offset in both inplane and crossplane directions, respectively.

The benefit of the chamber attached to the collimator is that it does not have to be positioned exactly at the collimator rotation axis, as long as it rotates together with collimator and is partially obscured by the half‐blocked jaw.

The ion chamber‐ and EPID‐based methods were performed in succession on four different linacs and the results compared.

## 3 RESULTS

### 3.A Reproducibility

Three linacs 1, 2, and 4 showed focal spot offsets less than 0.1 mm in each direction, but linac 3 had a significantly larger misalignment in the inplane direction, namely, 0.433 mm (Table 1).

**Table 1:** Reproducibility measurements of the 6MV focal spot offset using the EPID on four different Varian linacs

Measurement No.	Linac 1 (6EX)		Linac 2 (21iX)		Linac 3 (6EX)		Linac 4 (TrueBeam)	
	Crossplane	Inplane	Crossplane	Inplane	Crossplane	Inplane	Crossplane	Inplane
	(mm)	(mm)	(mm)	(mm)	(mm)	(mm)	(mm)	(mm)
1	−0.002	0.099	0.069	0.021	−0.027	−0.445	0.081	−0.020
2	−0.008	0.084	0.087	0.058	−0.026	−0.419	0.082	−0.018
3	−0.006	0.098	0.081	0.097	−0.032	−0.433	0.085	−0.046
Average	−0.005	0.094	0.079	0.059	−0.029	−0.433	0.083	−0.028
1SD	0.003	0.008	0.009	0.038	0.003	0.013	0.002	0.016

The average standard deviation (1 SD) of the focal spot offset for all linacs was 0.012 mm. However, high energy linacs (linac 2 and 4) showed increased relative uncertainty of source position offset in the inplane direction (Gun‐Target) compared to the crossplane direction.

### 3.B Independent validation

The ionization chamber validation method of the focal spot position agreed with the phantomless method with an average difference of 0.001 mm ± 0.015 mm (1 SD) (Table 2).

**Table 2:** Validation of the phantomless method of focal spot offset measurement with the ionization chamber method

Measurements	Linac 1 (6EX)		Linac 2 (21iX)		Linac 3 (6EX)		Linac 4 (TrueBeam)	
	Crossplane	Inplane	Crossplane	Inplane	Crossplane	Inplane	Crossplane	Inplane
	(mm)	(mm)	(mm)	(mm)	(mm)	(mm)	(mm)	(mm)
By ion chamber	−0.005	0.094	0.097	0.055	−0.036	−0.458	0.106	−0.024
By EPID	−0.005	0.094	0.079	0.059	−0.029	−0.433	0.083	−0.028
Difference	0.000	0.000	0.018	−0.004	−0.007	−0.025	0.023	0.004

## 4 DISCUSSION

A phantomless method has been developed for measuring the collimator treatment isocenter for both the jaws and MLC. When measurements are performed for both jaws and MLC, the difference in position of the isocenter centroids can be used as a measure of the alignment of the beam focal spot position with respect to collimator rotation axis. This is based on the principle that although the jaws and MLC share a common rotation axis, their different distances from the effective source position mean that their respective beam centers will project differently onto the EPID, if the radiation focal spot position is misaligned with collimator axis.

It is proposed that the test could be used as a quick monthly QA test of the beam focal spot position.

The monthly four‐field test provides a method whereby the linac's positional beam steering is checked in isolation. Traditional QA checks of beam profiles are often based upon large‐field measurement which are dominated by beam angle steering and often beam position steering is only checked via indirect means such as smaller field symmetry (e.g., 10 × 10 cm^2^) or by coarse methods such as X‐Ray vs. light‐field coincidence. This method presented provides a quick and accurate means of directly testing beam position steering, which not only makes position steering miscalibration easier to identify but also easier to rectify. This would enhance a department's ability to ensure a symmetric beam across the full range of field sizes and to ensure the correct geometric positioning of the beam penumbra.

The effective pixel size used for computation is 0.0392 mm, which was considered sufficient in this work. The precision of the calculations is, however, much finer, as the center of the field is calculated as an average of all pixels in the radiation field detected by EPID. No study was conducted to investigate the consequence of varying the effective pixel size on the accuracy of the method.

The reproducibility results show that the method is reproducible to the order of hundredths of a millimeter for the four linacs investigated for a test where tenths of a millimeter would be the clinically required level of accuracy.

Geometric analysis of the method shows that any inaccuracy of calibration of the EPID source‐imager distance marginally affects the uncertainty of the focal spot offset determination. For example, 1 mm error in the EPID source distance at the 100 cm level affects the determination of the distance between the two radiation isocenters by 0.1% (1 mm/1000 mm). Therefore, the uncertainty related to the EPID vertical calibration inaccuracy in calibration by 1 mm is only 0.001 mm, which is insignificant considering the uncertainty of the method itself (i.e., 0.012 mm). The method is also independent of the lateral and longitudinal positioning of an EPID, as only a relative difference in determined field centers is important.

The method has been validated by comparison with the department's ion chamber‐based method with a good agreement to within 0.025 mm across four linacs. This difference is clinically insignificant.

The focal spot position does vary with gantry angle, but this method is not appropriate for measuring this due to the effects of jaw and MLC sag at gantry angle other than 0° and 180°.

The fact that beam energies are position steered individually means that different beams can have different radiation isocenters. This has implications for the IsoCal calibration which is based entirely on the 6 MV beam. As such, IsoCal does not necessarily ensure alignment of imaging and treatment isocenters for all beams unless a method such as the one proposed in this study, among other things, is used to ensure that all beams have correct focal spot alignment. An option would be for Varian to include the test from this study (or a variant) in the new Machine Performance Check (MPC) application.^12^ Running MPC daily could then help ensure validity of the IsoCal calibration for all beam energies.

The Winston Lutz test currently has general acceptance for checking radiation to imaging isocenter alignment for Stereotactic QA. The test method of this study is not proposed as a replacement for Winston Lutz. The influences on radiation isocenter to imaging isocenter coincidence tested in Winston Lutz go beyond focal spot alignment. However, a regular test of focal spot alignment would remove this as a possibility for Winston Lutz fail and hence would help ensure consistency of the Winston Lutz results. Inplane focal spot position affects the position of radiation isocentre centroid and crossplane focal spot position affects radiation isocentre size and big enough changes in either of these radiation isocentre characteristics will result in a Winston Lutz fail. If a linac QA program also tests in isolation the other components that contribute to the Winston Lutz test, then it could be conceived that the Winston Lutz was no longer required or that at least the test could be performed at reduced frequency.

## 5 CONCLUSION

A novel, quick, easy, and accurate method has been presented for measuring radiation beam focal spot alignment using the EPID. The test provides a means of testing beam position steering, which is often not tested well in contemporary linac QA programs but is becoming more important with the advent of treatment techniques requiring high geometric accuracy such as CSRS and SBRT. The new technique is independently validated and is shown to be accurate and robust with reproducibility of 0.012 mm (1 SD) and could be used in conjunction with other tests to replace or minimize the need for regular Winston Lutz style tests, which are often quite time consuming and are difficult to diagnose the root cause of failure. It is recommended to perform this test regularly for all beam energies and this could ensure the validity of the IsoCal calibration for all beam energies.

## ACKNOWLEDGMENTS

We thank the anonymous reviewers' scrutiny of the manuscript and insightful critique.

## CONFLICT OF INTEREST

The authors declare no conflict of interest.
